# Straw and residual film management enhances crop yield and weakens CO_2_ emissions in wheat–maize intercropping system

**DOI:** 10.1038/s41598-021-93497-x

**Published:** 2021-07-07

**Authors:** Zhiwen Gou, Wen Yin, Qiang Chai

**Affiliations:** 1 College of Agronomy, Gansu Agricultural University, Lanzhou, 730070 China; 2 Gansu Provincial Key Laboratory of Aridland Crop Science, Lanzhou, 730070 China

**Keywords:** Environmental sciences, Agroecology, Climate-change ecology

## Abstract

Higher CO_2_ emissions and lower crop productivity are becoming thorny problems and restricted sustainable development of agriculture in arid inland areas. Intercropping has been shown to enhance crop productivity. However, Intercropping generally requires more input that led to an increase in CO_2_ emissions. It is unknown whether designing tillage and film mulching in reduction could decrease soil CO_2_ emissions in intercropping. Therefore, we integrated no tillage combined with residual film mulching and straw returning into wheat–maize intercropping. The maximal soil CO_2_ fluxes (F_s_) with intercropping was decreased by 12–21% compared to sole maize. Residual film mulching combined with straw returning (NTSMI) significantly reduced average F_s_ during the entire period of crop growth by 14–15%, compared with the conventional tillage (CTI). Soil CO_2_ emissions (CE) with intercropping was 18–20% less than that with sole maize and the NTSMI reduced CE by 12–16% compared to the CTI. The NTSMI boosted total grain yields (GY) by 14–17%, compared with the CTI. Wheat–maize intercropping significantly enhanced soil CO_2_ emission efficiency (CEE) by 33–41% in comparison to sole maize, and CEE with NTSMI was increased by 29–40% than that of CTI. A quadratic function for aboveground biomass (BA) combined with two linear functions for soil temperature (T_s_) and soil water-filled pore space (WFPS) was suitable for the monitored results. A multiple regression model composed of the above three factors can explain 73–91% of the F_s_ variation. Crop biomass accumulation at the time of maximal F_s_ was less with intercropping compared with sole maize. The structural equation indicated that the BA synergistic effect on CEE through combining negative effects on CE and positive effects on GY in intercropping. In conclusion, no tillage with straw returning and residual film mulching in wheat–maize intercropping was confirmed to be an optimum management practice to reducing soil CO_2_ emissions and enhancing soil CO_2_ emission efficiency in arid inland agroecosystem.

## Introduction

Globally, climate change due to increase greenhouse gas (GHG) emissions and food security are two main challenges around the world in the twenty-first century^1–3^. Thus, achieving high crop yields with minimal GHG emissions has become increasingly vital as a global solution to develop sustainable agriculture^4^. As one of the major sources of GHG emissions, agriculture contributed to GHG emissions approximately 20% to global climate change^5,6^. Reducing GHG emissions or increase GHG emission efficiency of agroecosystem is of great importance, one of the most effective strategies is to adopt appropriate farmland management practices^5,7–9^. Diversified cropping patterns and effective cultivation measures could decrease soil GHG emissions^10^ including intercropping patterns^11^, conservation tillage^12^, and crop residue management^13^.

Intercropping is a vital agronomic approach for sustainable intensification, it could produce high yields with simultaneous reduction in emissions^14–16^. In intercropping, previous researches have investigated soil CO_2_ emissions and production in relation to crops collocation^17^, water and fertilizer use management^1,2^, tillage measures and so on^14,18^. For instance, effect of crops with different traits were intercropped on carbon emissions reduction is different, researcher have observed that adopting to legume in maize-based intercropping could reduce soil CO_2_ emissions compared to cereal–cereal intercropping^11,19^. The effect of nutrient utilization indicated that intercropping decreased soil CO_2_ emissions via enhancing soil carbon and nitrogen storage^15,16^. Further, the decrease in carbon emissions in intercropping is reduced tillage coupled with straw returning^14,20,21^. Reduced tillage coupled with straw returning has become a vital practice in sustainable agricultural^9^. It was increasingly adopted for crop production due to environment friendly over conventional measures^22^. This technology can decrease soil temperature while retaining soil moisture during mid-season of crop growth, thereby effectively optimizing the relationship between crop growth and soil temperature and moisture^23,24^. In addition, numerous researchers have observed improved carbon sequestration under reduce tillage or straw returning^2,12,25,26^. Therefore, the development of reduce tillage and straw returning in intercropping is of great importance to soil CO_2_ emissions reduction in agroecosystem, which is one of the vital development directions in the future.

Wheat–maize intercropping has been generally applied in arid inland due to the increased demand for crop production, and it has made great contributions to grain production in this area^20,27^. Using film mulching in maize strips is an essential practice, it was widely adopted for inhibiting evaporation and increasing temperature in early spring when soil is cold in arid land^28^. Meanwhile, this practice can effectively inhibit weed, decrease leach of nutrients, accelerate plant growth and harvesting rainwater in rain fed, which is beneficial to increase crop yields^29,30^. However, soil temperature was increased by film mulching in mid-season crop growth can lead to accelerate crop senescence, thereby decreasing grain yields^31^. In addition, the balance of soil environmental conditions such as temperature and moisture was disturbed in a short duration^32^. It can lead to accelerate decomposition of soil organic matter, thereby enhancing soil respiration and soil CO_2_ emissions^30,33,34^. Obviously, reducing film input and development of environmentally friendly mulching technology are urgently needed for wheat–maize intercropping patterns in arid agroecosystem.

Previous researches have focused only on reducing CO_2_ emissions by improving tillage or straw management for wheat strip in wheat–maize intercropping system^14,20^, while ignoring the negative effects of film mulching for maize strip on environment. Actually, due to using new film mulching every year, the maize strip has higher CO_2_ emissions than that of the wheat strip in wheat–maize intercropping^21^. Therefore, it is vital to adopt advanced measures in both of wheat and maize strips, simultaneously. In our research, in view of the contradiction in crop growth of intercropped wheat and maize, enhancing soil CO_2_ emissions by new film mulching. On the basis of studies on improving wheat straw management for wheat strip in wheat–maize intercropping^14,23^, we proposed an innovation technology including no tillage combined with straw returning in wheat strips and residual film mulching in maize strips. In addition, little attention has been paid to integrated factors affecting soil respiration, such as dry matter accumulation and soil temperature and moisture. The effects of soil temperature, moisture and dry matter accumulation of crops on soil respiration were comprehensively considered, will contribute to exact estimate of soil carbon emissions. We hypothesized that integrating straw and residual film mulching into the intercropping pattern can achieve soil CO_2_ emissions reduction but enhance system productivity and soil CO_2_ emission efficiency. Furthermore, we consider the co-effects of soil temperature, soil moisture, and crop growth on soil CO_2_ emissions. The objectives of our research were to: (1) clarify the regulating effects of intercropping, no tillage, and mulching practice on soil CO_2_ emissions and crop productivity; (2) ascertain the responses of crop biomass accumulation and yield, soil moisture and temperature, and soil CO_2_ to different agronomic practices; (3) reveal the relationship between soil CO_2_ emissions of intercropping and soil moisture, soil temperature, and crop biomass accumulation. We further hypothesized that wheat–maize intercropping combined with residual plastic mulching and straw returning would decrease soil CO_2_ fluxes by regulating soil moisture, soil temperature, and crop biomass accumulation.

## Results

### Grain yield

**Figure 1:**
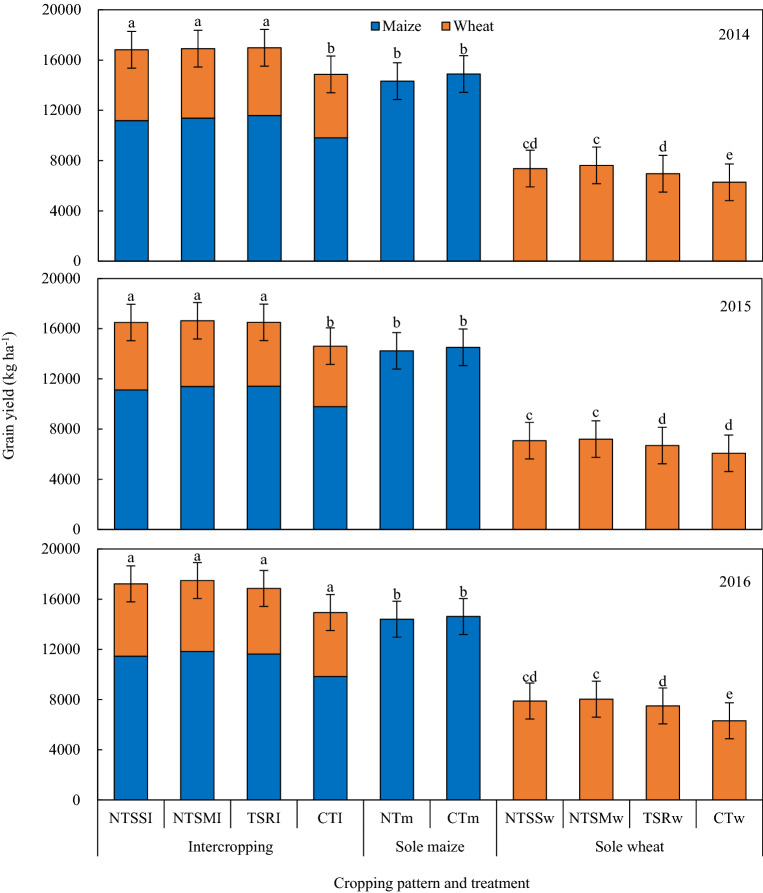
Grain yield of maize and wheat as affected by straw returning and film mulching in sole-crop and intercropping patterns in 2014, 2015, and 2016. Descriptions of treatment abbreviations are in Table 4. Different letters indicate significant differences (*P* ≤ 0.05) among treatments within a year. Error bars indicate standard errors of the means (n = 3).

Compared to sole cropping, intercropping coupled with wheat straw returning increased total grain yield significantly (Fig. 1). In 2014–2016, total wheat plus maize grain yield of intercropping increased by 15–20% and 124–138%, compared to sole maize and wheat, respectively. There was no significant difference in total grain yield between the CTI and sole maize cropping systems in 2014 and 2015. For the intercropping pattern, residual film mulching with straw returning boosted total grain yield, compared with the conventional tillage treatment; however, no significant difference in total grain yield across the three straw residue treatments. Compared to the CTI treatment, total grain yield was increased by 13–15%, 14–17%, and 13–14% with the NTSSI, NTSMI, and TSRI treatments, respectively. For sole cropping maize, tillage and different film mulching method did not significantly impact on grain yield. Straw returning significantly improved grain yield of sole wheat, increased by 17–25% under NTSSw, by 19–27% under NTSMw, and by 10–20% under TSRw in comparison with the CTw.

### Dynamics of soil CO_2_ fluxes

**Figure 2:**
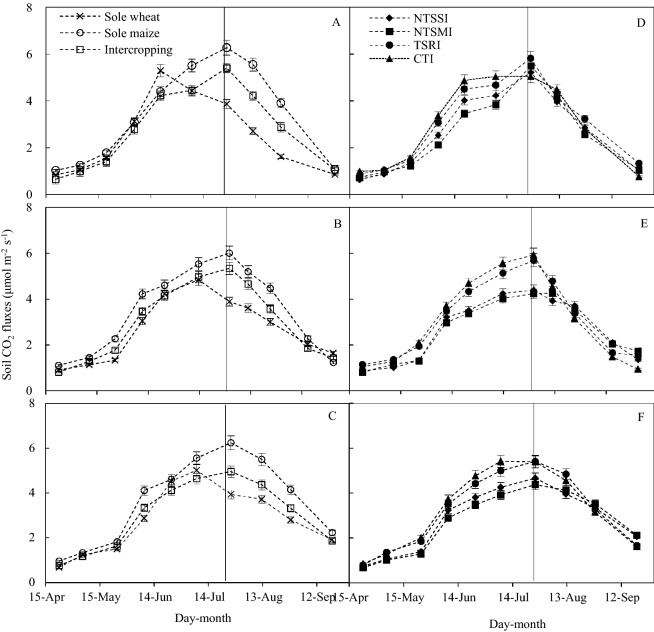
Seasonal variation of soil CO_2_ fluxes by cropping pattern, across treatments within a given cropping pattern (**A**–**C**), and straw management and film mulching treatment within wheat–maize intercropping (**D**–**F**) in 2014 (**A**, **D**), 2015 (**B**, **E**), and 2016 (**C**, **F**). Descriptions of treatment abbreviations are in Table 4. Error bars indicate standard errors of the means (n = 3). The vertical line in the middle part of each figure shows the date of wheat harvest.

Variation of soil CO_2_ fluxes (Fig. 2) was consistent with variation in air temperature in each year. Peak values of soil CO_2_ fluxes were observed in late-June to late-July and their time of occurrence was similar between sole maize and intercropping (Fig. 2A–C). Peak values of soil CO_2_ fluxes in both sole maize and intercropping appeared on the 26–31 July across the 3 years. Additionally, soil CO_2_ fluxes in all cropping patterns was decreased significantly after wheat was harvested. Compared to sole maize, soil CO_2_ fluxes after wheat harvest and maximal soil CO_2_ fluxes were significantly less with intercropping. In 2014–2016, maximal soil CO_2_ fluxes with intercropping was decreased by 12–21% compared to sole maize.

In intercropping, the different methods of straw management and film mulching significantly impacted on soil CO_2_ fluxes (Fig. 2D–F). In 3 years, the NTSSI and NTSMI treatments significantly reduced average soil CO_2_ fluxes during the entire period of crop growth by 11–14% and 14–15%, respectively, compared to the TSRI treatment, and by 11–15% and 10–16% in comparison with the CTI, respectively. Maximal CO_2_ fluxes had no significant difference among intercropping patterns in 2014; however, in the last 2 years, the NTSSI and NTSMI treatments significantly decreased maximal soil CO_2_ fluxes by a mean of 18% and 22% in comparison with the TSRI, respectively, and with a decrease of 20% and 24% in comparison with the CTI, respectively.

### Soil CO_2_ emissions during growing seasons

**Figure 3:**
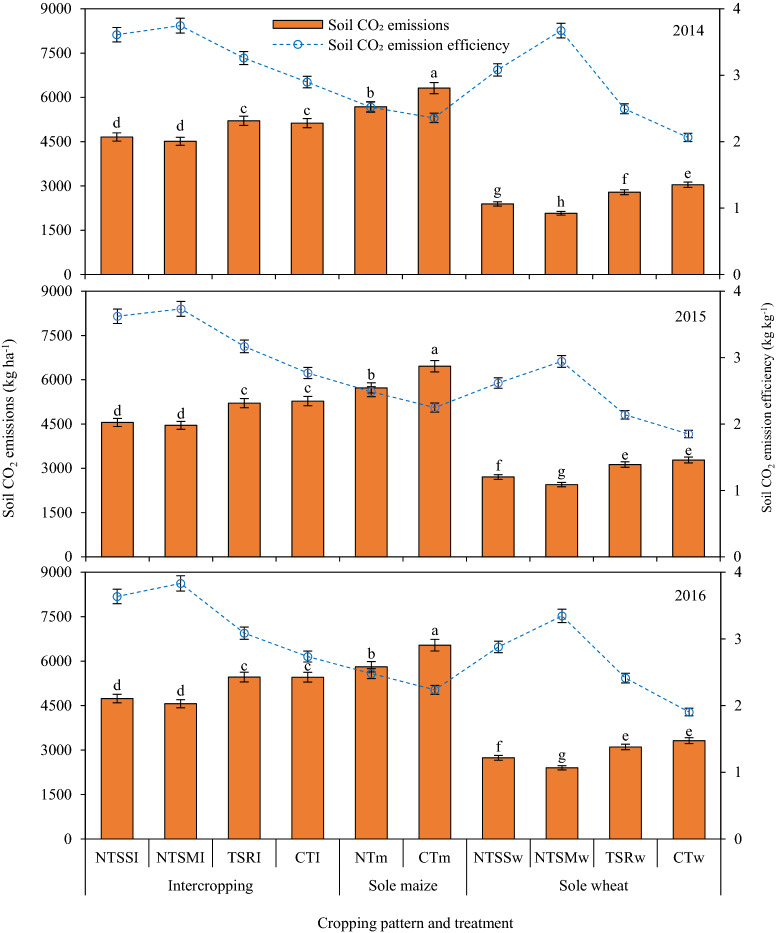
Effect of different straw returning and film mulching on soil CO_2_ emission and soil CO_2_ emission efficiency of maize and wheat in sole patterns and intercropping patterns in 2014–2016. The different letters are significantly different at 0.05 probability level, and the error bars indicate the standard errors of the means (n = 3). The descriptions of the treatment codes are given in Table 4.

During the growing season with intercropping was significant decreased soil CO_2_ emissions (CE) in comparison to sole maize; but compared with sole wheat, it can significant increased soil CO_2_ emissions (Fig. 3). Across treatments, CE with intercropping was 18–20% less than that with sole maize during the 3 years. Sole wheat emitted the least carbon, averaging 2573–2890 kg C ha^−1^ in 2014–2016. No tillage coupled with straw returning significantly decreased CE of sole wheat. With intercropping, CE did not differ significantly between the NTSSI and NTSMI treatments; however, they reduced CE by 12–13% and 13–17% in comparison to the TSRI treatment, respectively, and by a decrease of 9–14% and 12–16% in comparison to the CTI treatment, respectively. In sole wheat patterns, the NTSSw and NTSMw treatments decreased CE by 12–14% and 22–26%, respectively, compared to the TSRw treatment, and by 17–20% and 25–32%, respectively, compared to the CTw treatment. Additionally, CE differed significantly between the NTSSw and NTSMw treatments and was 10–13% less with the NTSMw in comparison with the NTSSw during the 3 years. For sole maize, residual film mulching (NTm) significantly decreased CE by 10–11%, compared with the conventional approach of tillage with annual new film mulching (CTm).

### Soil CO_2_ emission efficiency

Contrary to CE, intercropping significantly enhanced soil CO_2_ emission efficiency (CEE) in comparison to sole cropping (Fig. 3). Among years, average CEE across treatments with intercropping was 33–41% greater than that with sole maize and 20–32% greater than that with sole wheat. In intercropping, the NTSMI treatment significantly enhanced CEE in comparison to the TSRI and CTI treatments; however, there was no significant difference in soil CO_2_ emission efficiency between NTSMI and NTSSI. During the 3 years, CEE with NTSMI was 15–24% and 29–40% greater than that with TSRI and CTI, respectively. For sole wheat, the NTSMw treatment improved CEE by 12–19%, 38–47% and 57–78% during the 3 years, respectively, compared to the NTSSw, TSRw and CTw treatments. Similarly, the NTm treatment increased CEE by 7–11% in comparison to the CTm treatment in sole maize.

### Contribution of soil temperature and soil water-filled pore space to soil CO_2_ fluxes

The response of soil CO_2_ fluxes (F_s_) across growing seasons to soil temperature (T_s_) at the 0–10-cm layer was represented by an exponential equation (Table 1). This exponential model explained 28–52% of the variation in F_s_, depending on treatment. This relatively low and wide range indicates that F_s_ is a complex process and that additional variables may be needed to describe it. The temperature sensitivity of soil CO_2_ fluxes among treatments ranged from 1.682 and 2.460. Averaged across treatments, Q_10_ for sole maize, sole wheat and wheat–maize intercropping, was 1.734, 2.173, and 1.875, respectively. With intercropping, Q_10_ was 2.034 for the TSRI treatment, compared to 1.804–1.840 for the NTSSI, NTSMI and CTI treatments. A liner function was suitable for representing the relationship between soil water-filled pore space (WFPS) in the 0–30-cm layer and R_s_ across growing seasons (Table 2). This linear model accounted for 16–33% of the total variation in R_s_ and indicates that soil WFPS in the 0–30-cm layer may have had less of an individual influence on R_s_ than T_s_ at the 0–10-cm layer^11^.

**Table 1:** Parameter estimates for the exponential relationship between soil CO_2_ fluxes (F_s_, μmol m^−2^ s^−1^) and soil temperature (T_s_, °C) in the 0–10 cm depth across years by cropping treatment. F_s_ = *A* × *e*^*KTs*^.

Treatment^a^	Parameter estimate		Q_10_^b^	R^2^	P > F
	A^c^	K			
NTSSI	0.314	0.059	1.804	0.52	< 0.001
NTSMI	0.666	0.061	1.840	0.51	< 0.001
TSRI	0.327	0.071	2.034	0.50	< 0.001
CTI	0.404	0.060	1.822	0.45	< 0.001
NTm	0.305	0.058	1.786	0.29	0.002
CTm	0.306	0.052	1.682	0.28	0.002
NTSSw	0.290	0.090	2.460	0.35	0.001
NTSMw	0.361	0.067	1.954	0.49	< 0.001
TSRw	0.353	0.072	2.054	0.35	< 0.001
CTw	0.329	0.080	2.226	0.42	< 0.001

^a^Descriptions of treatment abbreviations are in Table 4.

^b^Q_10_: the rate of soil CO_2_ fluxes change with each 10 °C increase in soil temperature, calculated as *e*^*KTs*^*.*

^c^A and K are constants of the exponential equation.

**Table 2:** Functions and related parameters for the liner relationship between soil CO_2_ fluxes (F_s_) and soil WFPS (W_f_) at 0–30 cm depth in every cropping patterns: F_s_ = A × W_f_ + B.

Treatment^a^	Function parameter		*R*^*2*^	*P* > *F*
	A^b^	B		
NTSSI	0.139	− 2.901	0.33	0.001
NTSMI	0.121	− 2.610	0.30	0.001
TSRI	0.167	− 4.682	0.24	0.005
CTI	0.163	− 4.246	0.31	0.001
NTm	0.239	− 7.840	0.29	0.002
CTm	0.167	− 4.701	0.18	0.016
NTSSw	0.135	− 3.849	0.21	0.009
NTSMw	0.177	− 3.164	0.16	0.024
TSRw	0.146	− 4.413	0.27	0.003
CTw	0.123	− 2.688	0.17	0.023

The data were adopted to fit into the function in 2014–2016.

^a^The descriptions of the treatment codes are given in Table 4.

^b^A and B are constants of the liner equation.

### Co-effects of crop biomass accumulation, soil water-filled pore space, and temperature on soil CO_2_ fluxes

The co-effects of crop biomass accumulation, T_s_ at the 0–10-cm depth, and WFPS in the 0–30-cm dept on F_s_ across growing seasons were described using a quadratic function combined with two linear functions. Compared to regression models using only T_s_ or WFPS, this model significantly improved the prediction of F_s_, accounting for 73–91% of the total variation in F_s_ (Table 3). Using this model, estimates of crop biomass accumulation were calculated when soil CO_2_ fluxes was maximal. In sole wheat, peak values of F_s_ occurred when wheat biomass accumulation was 0.52–0.75 kg m^−2^, corresponding to 10–25 June when wheat was in the anthesis to early grain filling stages. Maximal F_s_ of sole maize occurred when maize biomass accumulation was 1.75–1.77 kg m^−2^, corresponding to 20 July to 7 August when maize was in the silking to early filling stages. The average of wheat and maize biomass accumulation with intercropping was less when F_s_ was maximal. Compared to sole maize, maximal F_s_ with intercropping occurred when the average of wheat and maize biomass accumulation was 1.32–1.51 kg m^−2^, corresponding to 10–20 July when wheat was in the grain filling stage and maize was in the early filling stage. Maximal F_s_ with intercropping occurred earlier and its duration was shorter, compared with sole maize. For intercropping, crop biomass accumulation at maximal F_s_ was greatest with the TSRI treatment. Compared to the TSRI treatment, crop biomass accumulation at maximal F_s_ with the NTSSI, NTSMI and CTI treatments was decreased by 8%, 4% and 13%, respectively.

**Table 3:** Relationship between crop biomass accumulation (BA: kg m^−2^), soil temperature (T_s_) and soil WFPS (W_f_) and soil CO_2_ fluxes in every cropping patterns: F_s_ = A × BA^2^ + B × BA + C × W_f_ + D × T_s_ + E.

Treatment	Function parameter					BA = − B/2A^a^ (kg m^−2^)	*R*^*2*^	*P* > *F*
	A^b^	B	C	D	E			
NTSSI	− 1.468	4.070	0.047	0.148	− 4.069	1.380	0.87	< 0.001
NTSMI	− 1.016	2.933	0.490	0.178	− 4.526	1.443	0.86	< 0.001
TSRI	− 1.354	4.080	0.051	0.293	− 6.282	1.507	0.91	< 0.001
CTI	− 1.892	4.984	0.055	0.211	− 5.955	1.317	0.88	< 0.001
NTm	− 1.540	5.454	0.043	0.112	− 3.120	1.771	0.73	0.006
CTm	− 1.173	4.633	0.064	0.135	− 4.911	1.750	0.90	< 0.001
NTSSw	− 0.933	1.396	0.014	0.203	− 1.934	0.748	0.83	< 0.001
NTSMw	− 1.668	1.949	0.019	0.219	− 1.293	0.584	0.83	< 0.001
TSRw	− 2.189	2.265	0.025	0.197	− 2.705	0.517	0.78	0.004
CTw	− 4.288	5.188	0.020	0.242	− 1.714	0.605	0.90	< 0.001

The data were adopted to fit into the function in 2014–2016.

^a^The amount of biomass accumulation when soil CO_2_ fluxes is maximal.

^b^The capital letters are related parameters of function.

^c^The descriptions of the treatment codes are given in Table 4.

### Relationships for the CE, GY, CEE between T_s_, WFPS and BA

**Figure 4:**
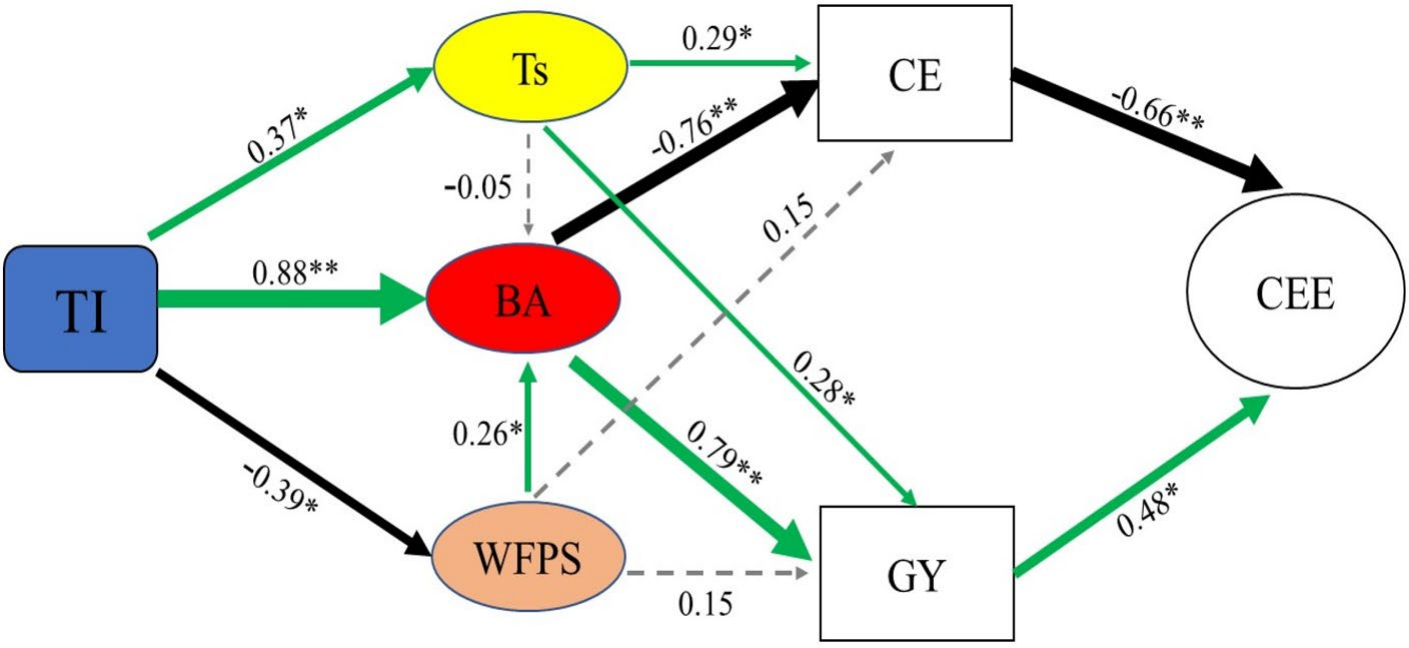
A structural equation of intercropping treatments effect on CEE, CE and GY in 2014–2016. The structural equation considered all possible pathways through soil temperature (Ts), soil water-filled pore space (WFPS) and crop biomass accumulation (BA) influence CEE, CE and GY in intercropping system. TI represents four intercropping treatments (NTSSI, NTSMI, TSRI, CTI). Green and black arrows represent significant positive and negative pathways, respectively. Grey dashed arrows indicate nonsignificant pathways. Arrow width is proportional to numbers indicate the standard path coefficients (SPC).

A structural equation model was used to determine the pathways of T_s_, WFPS and BA influencing the GY and CE to affect the CEE in intercropping system (Fig. 4). The result showed that across intercropping treatments (TI) had a direct positive effect on Ts and BA and intercropping treatments had a direct negative effect on WFPS. Among three factors, the Ts had a direct positive effect on CE and GY; The BA had a negative effect on CE and a positive effect on GY. Meanwhile, the CE had a negative effect on CEE and the GY had a positive effect on CEE. Overall, the structural equation indicated that the WFPS had no significant effect on CE and GY. The strength of the relationships for soil CO_2_ emissions and grain yields between crop biomass accumulation (SPC = − 0.76**, 0.79**) was greater than that they between soil temperature (SPC = 0.29*, 0.28*). In intercropping system, there were two main pathways of improving soil CO_2_ emissions efficiency (the pathway based on soil temperature and the pathway based on crop accumulation), and crop biomass accumulation is vital for it. The crop biomass accumulation increased soil CO_2_ emission efficiency mainly through its negative effect on soil CO_2_ emissions and the positive effect on grain yields.

## Discussion

### Yield advantage of straw and plastic managements in intercropping

The crop yield superiority of strip-intercropping compared to sole cropping has been shown in most studies^11,15,18^. This is attributed to improved resource utilization efficiency, due to spatial and temporal synchrony in the use of resources such as light, heat, and water by intercrops^11,35^. Intercropped crops have the competition of resources during the co-growth period partly, which it can undermines the growth of the late-maturing crop^36^. However, this adverse factor could be remedied by compensatory growth of the late-maturing crop after harvest of the early-maturing crop. The absorption zone for water and nutrients by the late-maturing crop can enlarge after the early-maturing crop harvest, thereby increasing the uptake of water and nutrients by the late-maturing crop and compensating for deficiencies at earlier stages^36^. For instance, dry matter accumulation of maize can be increased by compensation effect after wheat harvest in intercropping^37^. Wheat–maize intercropping has been increasingly adopted in areas with limited natural resources such as the northwestern China, most precipitation in this region occurs from July through September^38^, corresponding to the period of independent growth of maize after wheat harvest. Therefore, inhibiting evaporation after wheat harvest is vital for increasing yield of maize intercropped with wheat. In the present study, no tillage coupled with wheat straw mulching significantly increased total wheat plus maize grain yield in intercropping. This practice can inhibit evaporation^39^, and enhance soil moisture content in wheat–maize strip-intercropping^37^. It is beneficial to the most vigorous growth of maize after wheat harvest. Also, this advantage could be explained by niche differentiation, which made intercrops can utilize light and heat resources at different times in wheat–maize intercropping^29,38^. For conventional intercropping treatment, soil temperature and moisture were increased with annual new film mulching in maize strips, which also promotes early-season growth of maize and make the plant short of nutrients at late-season growth of maize^40^. It can cause senescence of plant and lead to decrease crop productivity. The contradiction between soil environment (soil temperature and moisture) and crop growth process can be coordinated effectively in intercropping wheat and maize through no tillage, combined with residual film mulching in maize strip and wheat straw mulching in wheat strip. Favorable soil moisture and temperature conditions could enhance grain filling and boost grain size of crops in intercropping.

### Soil CO_2_ fluxes and its controlling factors

In generally, soil CO_2_ fluxes is mainly determined by soil temperature and moisture, which can be affected by soil surface mulching management^41,42^. During the growing season, the soil CO_2_ fluxes varies with soil temperature, which is strongly associated with air temperature^43,44^. Soil CO_2_ fluxes values peaked in late-June to late-July in this study, corresponding to the time when soil temperature was greatest. An exponential model with soil temperature as the sole predictor variable explained 28–52% of the seasonal variation in soil CO_2_ fluxes. Soil moisture also has an effect on soil CO_2_ fluxes, but previous researches have confirmed no significant relationship between WFPS and soil CO_2_ fluxes^45^, perhaps due to a narrow range of WFPS. However, when the masking disturbance of soil temperature was accounted for, the response of soil CO_2_ fluxes to WFPS was improved^46^. This indicates that soil moisture and soil temperature can interact to influence soil respiration, and supports Ding et al.^45^, who reported that these factors usually change simultaneously and affect soil microbial activity. We found that the relationship between soil WFPS and soil CO_2_ fluxes could be described using a liner function, but this function explained only 16–33% of the season of growth variation in soil CO_2_ fluxes. In terms of the relationship between crops growth and soil CO_2_ fluxes, soil CO_2_ fluxes is consisted of releasing by rhizosphere respiration and basal respiration (soil organic matter derived CO_2_)^47^. In previous research, soil CO_2_ emitted by root respiration accounted for 27–76% of total soil CO_2_ fluxes^48^. Other studies found that increases in root biomass accumulation during the growing season enhanced root respiration and contributed to seasonal variation in soil respiration^46,49^. Meanwhile, aboveground growth of crops can directly affect root activity, since photo-assimilates provide the material basis for roots^19^. Therefore, the study of soil CO_2_ fluxes should consider the co-effects of soil temperature, soil moisture, and biotic factors^45,50^. Consistent with our hypothesis, a multiple regression model including soil temperature, soil WFPS, and aboveground biomass accumulation of crops accounted for 73–91% of the seasonal variation in soil CO_2_ fluxes. This model significantly improved the description of soil CO_2_ fluxes in comparison to soil temperature or WFPS alone. We believe that the difference, most like related to tillage and mulching management affecting crops biomass accumulation by regulating soil temperature and moisture, subsequently co-effecting soil CO_2_ fluxes.

Increases in crop biomass accumulation can intensify root respiration^11,19^. The growth of maize is reduced due to interspecific competition during crop symbiosis in intercropping^36,37^. For the above reasons, maximal soil CO_2_ fluxes was 12–21% less with intercropping in comparison to sole maize. Meanwhile, maximal soil CO_2_ fluxes occurred during the period of vigorous crop growth in the sole wheat and sole maize cropping patterns. The growth of intercropped maize was partially restricted by intercropped wheat at the stage of co-growth. The dry matter accumulation of maize in intercropping system was decreased compared to the maize in sole cropping system^36,37^. Also, crop biomass accumulation at the time of maximal soil CO_2_ fluxes was less with intercropping in comparison to sole maize. Additionally, the peak time of soil CO_2_ fluxes was earlier and shortened its duration in intercropping. These differences are attributed to film mulching in maize, which increased soil temperature, especially during early period of crop growth, and improved crop biomass accumulation. However, no tillage can reduce soil temperature and conserving soil moisture^51^. In this study, no tillage coupled with straw mulching in wheat and residual film mulching in maize decreased soil CO_2_ fluxes in intercropping, most likely because of the optimization of photo-assimilation for root respiration and soil moisture and decrease soil temperature^12^.

### Effects of straw and film management on soil CO_2_ emissions

Soil is the most important terrestrial carbon sink and crop production accounts for a large proportion of total carbon emission^52^. Hence, a vital approach to reduce soil CO_2_ emissions is the adoption of advanced technology for crop production^53^. Previous studies have shown that intercropping can effectively reduce soil CO_2_ emission in comparison to sole pattern^11,21^. Moreover, tillage is one of the most important sources of carbon emissions, and no tillage has the potential to sequester carbon due to a reduction in soil disturbance and increased soil organic carbon conservation^25,51,53^. Due to the effects of film mulching in intercropping system, the maize strip usually made a greater contribution to CO_2_ emission in comparison to wheat strip^21^. A key to reducing CO_2_ emission in maize-based intercropping is reduction of maize strips. For this purpose, we integrated that residual film mulching under no tillage and wheat straw returning into wheat–maize intercropping. We found that these measures could weaken soil CO_2_ emissions by a mean of 13% compared with conventional practices of tillage with annual new film mulched in maize strip and no straw returning under tillage in wheat strip, respectively. Similarly, residual film mulching has been shown to decrease soil temperature during early stages of maize development, delay growth of maize, and decrease root respiration of maize^14,22,54^. Additionally, annual new film mulching leads to high soil CO_2_ emissions by a greater roots biomass. Previous research showed that film mulching could increase root biomass by 104% in comparison to no mulching^55^. Thus, straw and residual film mulching could offset soil CO_2_ emissions. Wheat straw returning and residual film mulching coupled with intercropping significantly improved soil CO_2_ emission efficiency by weakening soil CO_2_ emissions and increasing total grain yields. Strip intercropping with wheat straw mulching in wheat, and residual film mulching in maize could coordinate the relationship between the soil environmental, crop growth, and soil CO_2_ fluxes, thereby enhancing crop productivity and low soil CO_2_ emission with reduced inputs in comparison to sole cropping and other intercropping patterns. Thus, this integrated approach is essential to reducing soil CO_2_ emissions and boosting crop productivity of arid agroecosystem.

## Conclusions

Average soil CO_2_ emissions with intercropping were 18–20% less than that of sole maize in 3 years. Wheat straw residual film mulching coupled with wheat–maize intercropping increased total grain yields of 14–17% compared to conventional intercropping (no straw returning and annual new film mulching). Additionally, average soil CO_2_ emission efficiency with intercropping was 33%–41% greater than that with sole maize and 20–32% greater than that with sole wheat. Compared to conventional intercropping treatment, residual film mulching coupled with wheat straw mulching in intercropping reduced soil CO_2_ emissions by 12–16% and enhanced soil CO_2_ emission efficiency by 29–40%. The improved mulching management optimized the relationship between soil temperature, soil moisture, and crop biomass accumulation to reduce soil CO_2_ fluxes with intercropping. Moreover, the peak time of soil CO_2_ fluxes was earlier and shortened its duration in intercropping. We conclude that residual film coupled with wheat straw mulching in intercropping is a vital approach to reducing soil CO_2_ emissions and intensifying crop productivity from arid inland agroecosystem. To achieve high crop yields with minimal GHG emissions to develop sustainable agriculture, the management of straw and residual film should be strengthened in arid inland agroecosystem. The findings of our study can provide a scientific and theoretical basis for establishing a less-emissions, high-yield and efficient cropping system in arid areas.

## Materials and methods

### Test site description

**Figure 5:**
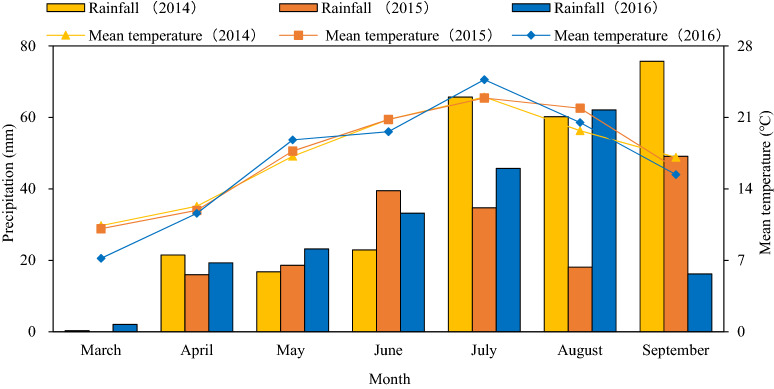
Monthly total precipitation and mean air temperature during the crop growth period in 2014, 2015, and 2016 at the Experimental station of Gansu Agricultural University in northwestern China.

Field experiments were carried out in 2014–2016 at the Huangyang Town (37° 34′ N, 102° 94′ E) in Wuwei City of northwestern China. In this place, the mean annual precipitation is approximately 200-mm (1960–2015); meanwhile, potential evaporation is over 2400-mm and rainfall is concentrated in late July through October (Fig. 5). Thus, crop production relies on irrigation. Long-term annual averaged air temperature is 7.3 °C, and accumulated air temperature greater 10 °C is approximately 3000 °C. This experimental site is representative of the land in arid inland agroecosystem.

### Experimental design and crop management

**Figure 6:**
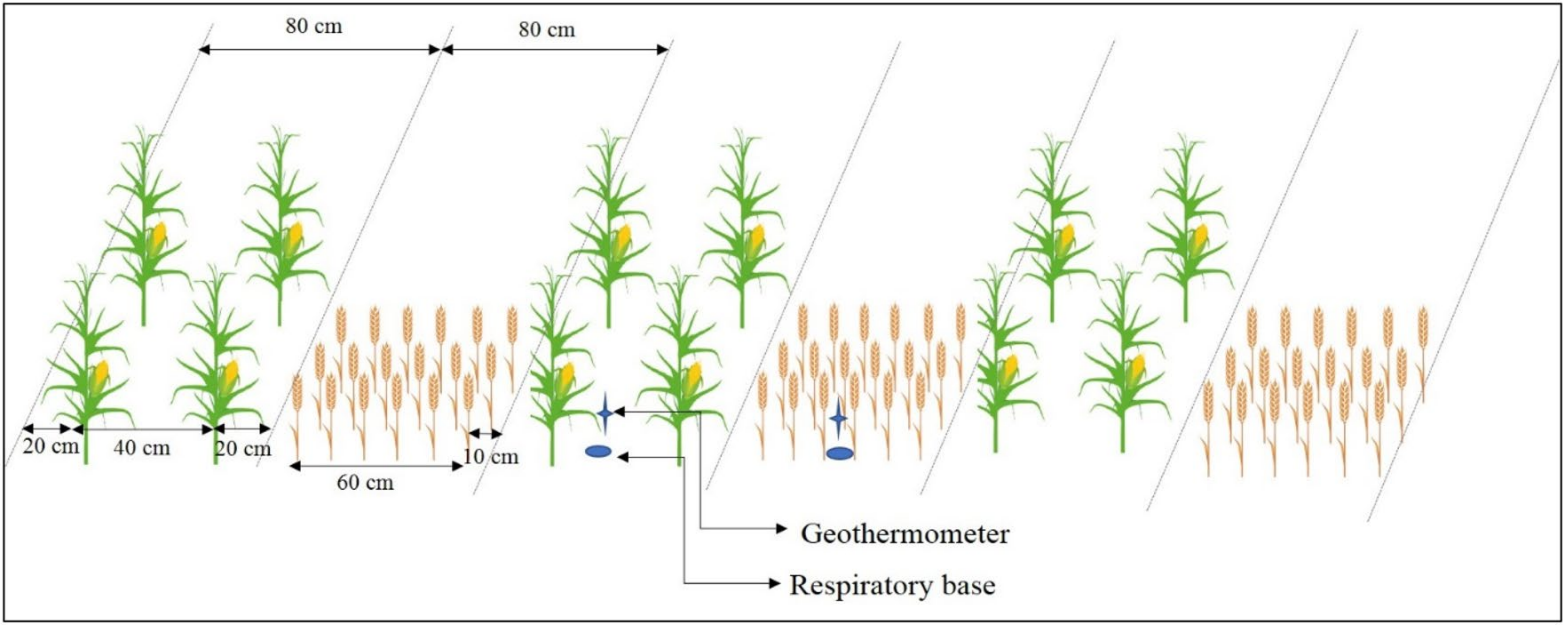
Layout of wheat–maize intercropping pattern and the field locations of the geothermometer and respiratory base in each study year.

A randomized complete block design (RCBD) was used in the study to test different ten treatments with repeat of three times (Table 4). Each plot (10 m long and 4.8 m wide) was surrounded by 60-cm width ridges to prevent surface runoff and subsurface lateral infiltration. Treatments were applied to the same plots each year and the cropping patterns in 2013 were the same as those during the experiment. Tillage, and mulching practices were imposed in 2013 according to the experimental treatments. This study evaluated sole maize, sole wheat, and wheat–maize strip intercropping, each with two tillage methods (no tillage and conventional tillage). Four wheat straw managements that application to sole wheat and the wheat strips of the intercropping pattern at wheat harvest in last summer, and included wheat straw standing with no tillage (wheat straw was chopped at 25–30 cm above the soil surface), wheat straw mulching with no tillage (25–30 cm tall of wheat straw was chopped evenly mulching on the soil surface), incorporation of wheat straw to the soil with conventional tillage (25–30 cm tall of wheat straw was chopped evenly), and no straw returning under conventional tillage. Residual film mulching with no tillage and annual new film mulching with tillage after maize harvest were evaluated in sole maize and the maize strips in the intercropping pattern (Table 4). The depth of all tillage operation is 30-cm. The film is colorless and transparent with 0.008-mm thickness and it was applied to maize strip before sowing in spring. The width of both wheat and maize strips is 80 cm of intercropping. It was planted at strips of two rows for maize alternated with six rows for wheat, with three pairs of intercrops strips arranged in each intercropped plot. The row spacing of wheat and maize is 12-cm and 40-cm, respectively (Fig. 6).

**Table 4:** Description of treatments.

Treatment abbreviation	Cropping pattern	Tillage method	Mulching method	
			Maize strip^a^	Wheat strip^b^
NTSSI	Intercropping	No tillage	Residual film mulching	25–30 cm tall straw standing
NTSMI				25–30 cm tall straw mulching
TSRI		Conventional tillage	Annual new film mulching	25–30 cm tall straw incorporated into soil
CTI^c^				No straw returning
NTm	Sole maize	No tillage	Residual film mulching	
CTm		Conventional tillage	Annual new film mulching	
NTSSw	Sole wheat	No tillage		25–30 cm tall straw standing
NTSMw				25–30 cm tall straw mulching
TSRw		Conventional tillage		25–30 cm tall straw incorporated into soil
CTw				No straw returning

^a^Residual plastic mulching: e.g., 2-years film mulching: soil was mulched with new film in the previous year and the plastic was preserved with no tillage and sowing directly on the residual plastic in the following spring. Annual new film mulching: after maize harvest in the previous year, residual film was removed and soil was tilled, and in the following spring soil was mulched with new film and maize was sown.

^b^Straw management was implemented following wheat harvest in the previous year.

^c^Conventional intercropping treatment (CTI): wheat straw cut above the soil surface and removed it before conventional tillage in wheat strip and annual new film mulching in maize strips. Wheat straw in CTI and CTw treatment and maize straw in all treatments were removed out of fields.

The irrigation rate, fertilizer rates, sowing date, and harvesting date of crops in sole and intercropping patterns are in Table 5. Maize (*cv. Xian-yu 335*) and wheat (*cv. Ning-chun 2*) were planted in each year and had the same sowing and harvesting dates in sole and intercropping patterns. The same area-based rate of fertilizer was applied for a given crop in sole crop and intercropping patterns. Nitrogen and phosphorus fertilizers were applied using urea and diammonium phosphate, respectively. All fertilizer N and P for wheat and all fertilizer P for maize were top-dressed before sowing. For maize, fertilizer N was applied at three times, with 30%, 60%, and 10% of the total top-dressed prior to sowing, at the six-leaf collar maize phenological stage, and at the kernel blister maize phenological stage, respectively. Irrigation water was applied using drip irrigation. The use of maize and wheat seeds in the present study was permitted by Gansu Agricultural University and it complies with local and national guidelines and legislation.

**Table 5:** Irrigation rate, fertilizer rate, sowing date, and harvest date of crops in sole and intercropping patterns, in 2014, 2015, and 2016.

Crop	Irrigation rate (m^3^ ha^−1^)	Fertilizer rate (kg ha^−1^)		Sowing date			Harvest date		
		N	P_2_O_5_	2014	2015	2016	2014	2015	2016
Intercropped maize	6000	225	113	4/25	4/24	4/20	10/1	9/28	9/20
Intercropped wheat		113	75	3/21	3/29	3/30	7/24	7/28	7/21
Sole maize	5250	450	225	4/25	4/24	4/20	10/1	9/28	9/20
Sole wheat	3600	225	113	3/21	3/29	3/30	7/24	7/28	7/21

### Measurement and calculation Aboveground biomass accumulation and grain yield

Aboveground biomass accumulation (BA) was monitored at the main growth stages of intercrops. For the intercropped plots, one pair of wheat and maize strips was used to monitor aboveground biomass accumulation and the remaining two pairs of wheat and maize strips were used to monitor grain yield at physiological maturity. In sole-cropped plots, one-half of the plot was used to monitor aboveground biomass accumulation and the other half was used to monitor grain yield at physiological maturity. At each sampling time, aboveground biomass accumulation was monitored from 20 wheat and 10 maize plants in the same row that were randomly sampled and cut at the soil surface. Plant samples were dried in a forced-air oven for 30 min at 105 °C, and then at 80 °C until changeless mass. Plant samples weighed on an electronic balance. Grain yield (GY) of wheat and maize was measured when at crops reached physiological maturity. Ears of wheat and maize were hand-harvested from all plants in two pairs of crop strips in intercropped plots and one-half of sole-cropped plots, air dried, threshed, and weighed. Grain yield of each crop was determined based on the air-dried weight obtained for a given plot.

### Soil temperature

Soil temperature (T_s_, °C) at the 0–10-cm soil layer in each plot was measured adopt a curved pipe geothermometer (Wuqiang Regong Meter Plant, Hebei China). In the intercropped plots, two thermometers were placed in the central of each intercropped strip (Fig. 6), and the mean of the two values was suitable for representing each plot. One thermometer was placed in each of sole cropping. Soil temperature was measured on 3-day intervals during the entire period of crop growth at 8:00, 14:00, and 18:00 on each day of measurement. The calculate of soil temperature is the average of three time points per day.

### Soil water content

Gravimetric soil water content (W_g_) of the 0–30-cm soil depth was monitored in 10-cm increments adopting the oven-drying method every 20 days during the entire period of crop growth. Two measuring values were taken from each of intercropped strips in the intercropping treatments and one value was taken from each of sole treatment. Soil water-filled pore space (WFPS) was determined by the formula as follows:

1 $${\text{WFPS}} = {\text{W}}_{{\text{g}}} \times {\text{BD}} \times \frac{{{\text{PD}}}}{{{\text{PD}} - {\text{BD}}}}$$

where W_g_ (%) is gravimetric soil water content, BD (g cm^−3^) is soil bulk density, and PD (g cm^−3^) is particle density of soil with a value of 2.65 g cm^−3^^45^.

### Soil CO_2_ fluxes

Soil CO_2_ fluxes (F_s_) was measured in each plot at major growth stages using a LI-8100A system (LI-COR, 4647 Superior Street Lincoln, Nebraska USA). Soil CO_2_ fluxes was measured at every 2 h during each day of measurement from the center of each sole-cropped plot and each intercropped strip in every intercropping plot (Fig. 6). Soil CO_2_ fluxes of intercropped plots was calculated as the mean of wheat and maize strips. Before measuring CO_2_ fluxes (prior to seeding), the respiratory base was pushed 2–3-cm into the soil, and film mulch and other crop residues were removed from the location of the respiratory base in maize, there was no film and residues for the whole growth stage.

The relationship between soil CO_2_ fluxes with soil temperature was represented by an exponential function:

2 $${\text{F}}_{{\text{s}}} = {\text{A}} \times {\text{e}}^{{{\text{KTs}}}}$$

where F_s_ is soil CO_2_ fluxes, Ts is soil temperature at the 0–10-cm soil layer, A and K are constants of the function.

Temperature sensitivity (Q_10_) is calculated as e^KTs^ and is the rate of soil CO_2_ fluxes change with peer 10 °C increase in soil temperature ^19^.

The relationship between soil CO_2_ fluxes with WFPS was represented by a liner function:

3 $${\text{F}}_{{\text{s}}} = {\text{A}} \times {\text{W}}_{{\text{f}}} + {\text{B}}$$

where W_f_ is soil water-filled pore space (WFPS) in the 0–30-cm layer, A and B are constants of the function.

A quadratic function combined with two linear functions was suitable for representing the relationship among soil CO_2_ fluxes and crop biomass accumulation, soil temperature, and WFPS as follows:

4 $${\text{F}}_{{\text{s}}} = {\text{A}} \times {\text{BA}}^{{\text{2}}} + {\text{B}} \times {\text{BA}} + {\text{C}} \times {\text{W}}_{{\text{f}}} + {\text{D}} \times {\text{T}}_{{\text{s}}} + {\text{E}}$$

where BA, Wf, and Ts are crop biomass accumulation, soil water-filled pore space, and soil temperature, respectively, and A, B, C, D, and E are constants of the function.

### Soil CO_2_ emissions and CO_2_ emission efficiency

Soil CO_2_ emissions (CE) was calculated is based of soil CO_2_ fluxes (F_s_) adopting the following formula expressed by^11^.

5 $${\text{CE}} = \sum \left[ {\frac{{{\text{F}}_{{{\text{s}}_{{{\text{i}} + 1}} }} + {\text{F}}_{{{\text{s}}_{{\text{i}}} }} }}{2}\left( {{\text{t}}_{{{\text{i}} + 1}} - {\text{t}}_{{\text{i}}} } \right) \times 0.1584 \times 24} \right] \times 0.2727 \times 10$$

where F_s_ is soil CO_2_ fluxes (µmol CO_2_ m^2^ s^−1^), *i* + 1 and *i* are the current and the last monitoring date, respectively, *t* is days after sowing stage, 0.1584 converts mol CO_2_ m^−2^ s^−1^ to g CO_2_ m^−2^ h^−1^, and 0.2727 converts g CO_2_ m^−2^ h^−1^ to g C m^−2^ h^−1^.

Soil CO_2_ emission efficiency (CEE) was calculated using grain yields and soil CO_2_ emissions as follows^14^:

6 $${\text{CEE}} = \frac{{{\text{Grain}}\;{\text{yield}}\;({\text{kg}}\;{\text{ha}}^{{ - 1}} )}}{{{\text{Carbon}}\;{\text{emissions}}\;({\text{kg}}\;{\text{ha}}^{{ - 1}} )}}$$

### Statistical analysis

One-way analysis of variance (ANOVA) followed by the Duncan’s multiple-range test was performed to determine the effects of treatment for each year using SPSS 20.0 (SPSS Institute Inc, USA). The significances among treatments were presented at *P* < 0.05. The relationships among soil temperature, soil moisture and biomass accumulation and soil CO_2_ fluxes were determined by nonlinear regression analyses. The pathways of how soil and crop growth factors influencing the grain yields and soil CO_2_ emissions to affect the soil CO_2_ emission efficiency in intercropping system were explored by a structural equation model. This model was determined using R version 3.2.0 (R Foundation for Statistical Computing, Vienna, Austria, 2015).
